# Statin Utilization for Primary Prevention of Cardiovascular Disease in Patients Developing First ST‐Segment Elevation Myocardial Infarction: A Malaysian Retrospective Cohort Study

**DOI:** 10.1002/hsr2.70206

**Published:** 2025-03-02

**Authors:** Abdullah Faiz Zaihan, Shairyzah Ahmad Hisham, Aina Yazrin Ali Nasiruddin, Chia Siang Kow, Syed Shahzad Hasan, Dinesh Sangarran Ramachandram

**Affiliations:** ^1^ Department of Pharmacy Shah Alam Hospital Shah Alam Selangor Malaysia; ^2^ Faculty of Pharmacy University of Cyberjaya Cyberjaya Selangor Malaysia; ^3^ School of Pharmacy University of Nottingham Malaysia Semenyih Selangor Malaysia; ^4^ School of Pharmacy IMU University Kuala Lumpur Federal Territory of Kuala Lumpur Malaysia; ^5^ School of Applied Sciences University of Huddersfield Queensgate Huddersfield UK; ^6^ School of Pharmacy Monash University Malaysia Subang Jaya Selangor Malaysia

**Keywords:** acute coronary syndrome, diabetes, HMG‐CoA reductase inhibitor, lipid lowering, prophylaxis

## Abstract

**Background and Aims:**

This retrospective cohort study aimed to determine the utilization rate of statin therapy for primary prevention of cardiovascular disease (CVD) and factors associated with its use in patients prior to experiencing their first ST‐elevation myocardial infarction (STEMI).

**Methods:**

The study included 177 patients admitted for their first episode of STEMI at a Malaysian secondary hospital in 2020. Data were retrospectively collected from the electronic health information system. Logistic regression analysis was performed to identify significant predictors of statin therapy utilization for primary prevention.

**Results:**

Of the 177 patients, only 15.8% (*n* = 28) had been prescribed statin therapy for the primary prevention of CVD prior to their first STEMI. Hypertension and dyslipidemia were identified as significant predictors of statin use, with adjusted odds ratios (AOR) of 9.570 (95% CI: 1.859–49.281) and 37.900 (95% CI: 6.716–213.87), respectively. Follow‐up data indicated significantly reduced cholesterol levels post‐STEMI, demonstrating the lipid‐lowering effectiveness of statin therapy.

**Conclusion:**

This study highlights the low utilization rate of statin therapy for primary prevention of CVD among patients prior to their first STEMI. Despite the proven effectiveness of statins in lowering cholesterol levels post‐STEMI, there is a need to enhance awareness, adherence to guidelines, and efforts to achieve LDL‐C targets through appropriate statin therapy.

## 1 Introduction

The term cardiovascular disease (CVD) encompasses a wide range of conditions that affect the heart and blood vessels, including coronary heart disease (CHD), cerebrovascular disease, peripheral arterial disease, rheumatic heart disease, congenital heart disease, and venous thromboembolism [1]. According to the World Health Organisation (WHO), CVD remains the leading cause of mortality globally, accounting for approximately 17.9 million deaths annually [2]. In Malaysia, CHD is the primary contributor of death, responsible for nearly 17% of medically certified deaths in 2020 [3, 4].

Coronary heart disease (CHD) is characterized by the narrowing or blockage of coronary arteries due to plaque buildup, which compromises blood flow and oxygen delivery to the heart. A rupture of the plaque or the formation of a blood clot within the coronary arteries can precipitate acute myocardial ischemia, a condition that, if untreated, culminates in myocardial infarction (MI). Among the types of MI, ST‐elevation myocardial infarction (STEMI) is particularly severe, resulting from a complete occlusion of a coronary artery and affecting a significant portion of the heart muscle.

Extensive research has identified major risk factors contributing to CHD, many of which are modifiable with early intervention. The Framingham Heart Study established traditional risk factors, including hypertension, diabetes, cigarette smoking, dyslipidemia, obesity, and physical inactivity [5, 6]. Similarly, The Effect of Potentially Modifiable Risk Factors Associated with Myocardial Infarction (INTERHEART) study identified nine modifiable risk factors for acute MI across diverse populations, including abnormal lipid profiles, smoking, hypertension, diabetes, abdominal obesity, psychosocial stress, poor dietary habits, alcohol consumption, and inadequate physical activity. Together, these studies underscore the importance of addressing modifiable risk factors to mitigate the risk of CHD and MI. [7].

Pharmacological interventions, particularly statins, are pivotal for managing dyslipidemia and reducing the risk of CHD and MI. Statins are well‐established as a cornerstone therapy for the primary prevention of CVD, with overwhelming evidence supporting their efficacy in lowering low‐density lipoprotein cholesterol (LDL‐C) and reducing cardiovascular events. Despite clear recommendations in clinical practice guidelines (CPGs), the real‐world utilization of statin therapy remains suboptimal, particularly among high‐risk populations [8, 9, 10, 11]. This gap in implementation contributes to preventable first MI events, imposing significant burdens on patients, families, and healthcare systems.

In Malaysia, there is a lack of published studies investigating the relationship between cardiovascular risk factors, prior statin use, and the incidence of first STEMI. Additionally, the extent of adherence to the Malaysian CPGs for dyslipidemia and cardiovascular disease management, introduced five years ago, remains unclear. This study aimed to evaluate the utilization of statin therapy for the primary prevention of CVD and to identify factors associated with its use among patients before their first STEMI. This investigation addresses a critical gap in the understanding of statin therapy implementation and its impact on cardiovascular outcomes in the Malaysian context.

## 2 Methods

### 2.1 Study Design

This study employed a retrospective cohort design conducted between June and August 2022. Data were sourced from the electronic healthcare information system (eHIS) of Shah Alam hospital, a secondary hospital in Selangor, Malaysia. This study reviewed electronic medical records of patients admitted for their first ST‐elevation myocardial infarction (STEMI) episode in 2020. Data extraction covered the entire year of 2020, with information retrieved from eHIS and transferred into a standardized data collection form. To protect patient privacy and confidentiality, all data sets were anonymized.

### 2.2 Patient Selection

The study included adult patients aged 18 years and above with a primary documented diagnosis of first STEMI admitted to Shah Alam Hospital between January 1, 2020, and December 31, 2020. Patients transferred to other hospitals after fibrinolysis and those with incomplete or missing data were excluded.

### 2.3 Data Collection

Data were collected retrospectively from the eHIS of Shah Alam Hospital. Researchers underwent training before data collection commenced to ensure accuracy and consistency. The data collection form, developed by the research team based on relevant CPGs and previous studies was structured into four sections to capture all necessary information:

1. Demographics: Included age, gender, ethnic group, admission and discharge dates, baseline blood pressure, and heart rate.

2. CVD Risk Factors: Documented underlying conditions, smoking history, family history of CVD, and Framingham General CVD Risk Score. Hypertension was defined as a history of elevated blood pressure or antihypertensive medication use, while dyslipidemia was defined as a history of abnormal lipid levels or use of lipid‐lowering medications.

3. Lipid Profile: Recorded baseline total cholesterol (TC) and low‐density lipoprotein cholesterol (LDL‐C) levels upon STEMI admission and 3‐month post‐STEMI values. Reference ranges for TC and LDL‐C were based on Malaysian CPGs.

4. Statin Therapy: Included data on statin use before STEMI, type of statin therapy, statin use at discharge, and the type of statin prescribed.

Baseline TC and LDL‐C levels were defined as values measured upon admission for the first STEMI episode, while post‐STEMI values were defined as levels measured at least 3 months post‐STEMI. Reference ranges for TC and LDL‐C target levels were adopted from the Malaysian CPG. The Framingham General CVD risk assessment tool was used to assess 10‐year CVD risk, with risk classification following recommendations in the Malaysian CPG.

### 2.4 Data Analysis

Statistical analyses were conducted using IBM SPSS Statistics version 26.0. Normality of continuous variables was assessed through skewness and kurtosis, with values between −2 and +2 considered indicative of normal distribution [12]. Descriptive statistics were applied to summarize data: means and standard deviations for normally distributed continuous variables, and frequencies and percentages for categorical variables.

Inferential analyses included paired *t*‐tests to evaluate associations between variables. Logistic regression analysis was performed to assess the relationships between demographic and clinical characteristics and the use of statin therapy for primary prevention of CVD. Univariate regression analysis identified significant variables, which were subsequently included in multivariate regression analysis to determine independent predictors. Results were presented as odds ratios (OR) with 95% confidence intervals (CI). All statistical tests were two‐tailed, with significance set at *p* < 0.05.

## 3 Results

A total of 212 patients admitted to Shah Alam Hospital for their first episode of STEMI between January 1 and December 31, 2020, were initially screened for eligibility according to the study's inclusion and exclusion criteria. Among these, 35 patients were excluded due to missing or incomplete data, resulting in a final study cohort of 177 patients (Table 1).

**Table 1:** Demographic and baseline characteristics of patients included in the study (*N* = 177).

Characteristics	*n*	Percentage (%) or mean (SD)
Sex		
Male	158	89.3
Female	19	10.7
Age, year		52.7 (12.0)
Race		
Malay	102	57.6
Chinese	13	7.3
Indian	34	19.2
Others	28	15.8
Baseline systolic blood pressure, mmHg		136.6 (21.7)
Baseline diastolic blood pressure, mmHg		84.1 (15.8)
Diabetes	85	48.0
Hypertension	70	39.5
Dyslipidemia	71	40.1
History of smoking	108	61.0
Family history of CVD	35	19.8
On statin before STEMI	28	15.8
Baseline TC, mmol/L		5.13 (1.16)
Baseline LDL‐C, mmol/L		3.24 (1.06)
a10‐year CVD risk, %		27.6 (18.7)

Abbreviations: CVD, cardiovascular disease; LDL‐C, low‐density lipoprotein cholesterol; SD, standard deviation; STEMI, ST‐elevation myocardial infarction; TC, total cholesterol.

^a^ As estimated by the Framingham general CVD risk assessment tool.

### 3.1 Demographic and Clinical Characetristics of Study Subjects

The majority (89.3%, *n* = 158) of patients were male, with a mean (SD) age of 53 (12.0) years. The largest ethnic group was Malay (57.6%, *n* = 102), followed by Indian (19.2%, *n* = 34). Nearly half of the patients had diabetes (48.0%, *n* = 85), while 39.5% (*n* = 70) and 40.1% (*n* = 71) of patients had hypertension and dyslipidemia, respectively. A family history of cardiovascular disease (CVD) was reported by 19.8% (*n* = 35) of the patients. Smoking emerged as the most prevalent CVD risk factor, with 61.0% (*n* = 108) of the patients reporting a history of smoking. The baseline lipid profile of the study cohort is summarized in Table 1, with mean (SD) baseline TC and LDL‐C levels recorded at 5.13 (1.16) mmol/L and 3.24 (1.06) mmol/L, respectively.

### 3.2 Use of Statin Therapy Prior to First STEMI

The characteristics of the study cohort, stratified by the use statin therapy for primary prevention of CVD prior to their first STEMI, are presented in Table 2. Only 15.8% (*n* = 28) of patients were prescribed statin therapy prior to their first STEMI. Among these patients, the majority had comorbidities such as diabetes (78.6%, *n* = 22), hypertension (89.3%, *n* = 25), and dyslipidemia (89.3%, *n* = 25). In contrast, patients not on statin therapy exhibited a higher prevalence of smoking and family history of CVD.

Baseline total cholesterol (TC) and low‐density lipoprotein cholesterol (LDL‐C) levels were notably lower in patients receiving statin therapy compared to those without prior statin use (4.52 mmol/L vs. 5.24 mmol/L and 2.52 mmol/L vs. 3.37 mmol/L, respectively). The mean (SD) 10‐year CVD risk was significantly higher among patients who received statin therapy for primary prevention before their first STEMI [37.1% (24.7) vs. 25.8% (16.9)].

**Table 2:** Baseline characteristics of patients stratified by the use of statin therapy prior to first STEMI.

Characteristics	Statin use Before STEMI (*n* = 28)	No statin use before STEMI (*n* = 149)
Diabetes	22 (78.6%)	63 (42.3%)
Hypertension	25 (89.3%)	45 (30.2%)
Dyslipidaemia	25 (89.3%)	46 (30.9%)
History of smoking	10 (35.7%)	98 (65.8%)
Family history of CVD	2 (7.1%)	33 (22.1%)
Baseline TC, mmol/L	4.52 (1.16)	5.24 (1.13)
Baseline LDL‐C, mmol/L	2.52 (1.08)	3.37 (1.00)
a10‐year CVD risk, %	37.1 (24.7)	25.8 (16.9)
CV risk categories		
Low‐risk	3 (10.7%)	23 (15.4%)
Intermediate‐risk	7 (25.0%)	45 (30.2%)
High‐risk	18 (64.3%)	81 (54.4%)

Abbreviations: CVD, cardiovascular disease; LDL‐C, low‐density lipoprotein cholesterol; STEMI, ST‐elevation myocardial infarction; TC, total cholesterol.

^a^ As estimated by the Framingham general CVD risk assessment tool.

Of the 28 patients who prescribed statin therapy for the primary prevention of CVD prior to their first STEMI, the majority (89.3%, *n* = 25) received moderate‐intensity statin therapy, while only 10.7% (*n* = 3) were prescribed high‐intensity statin therapy. Simvastatin was the most frequently prescribed statin for primary prevention (53.6%, *n* = 15), followed by atorvastatin (42.8%, *n* = 12), with rosuvastatin being the least commonly prescribed (3.6%, *n* = 1).

### 3.3 Factors Associated With the Prescription of Statin Therapy for Primary Prevention of CVD Prior to First STEMI

Univariate analyses identified several factors significantly associated with the use of statin therapy for the primary prevention of CVD prior to the first STEMI. These factors included age (OR: 1.06; 95% CI: 1.02–1.10), male sex (OR: 0.20; 95% CI: 0.07–0.56), diabetes (OR: 5.01; 95% CI: 1.92–13.07), hypertension (OR: 19.26; 95% CI: 5.53–67.06), dyslipidemia (OR: 18.66; 95% CI: 5.36–64.93), and chronic kidney disease (OR: 10.58; 95% CI: 2.37–47.29).

Conversely, a history of smoking (OR: 0.29; 95% CI: 0.12–0.67), higher baseline total cholesterol (OR: 0.53; 95% CI: 0.35–0.81), and higher baseline LDL‐C levels (OR: 0.39; 95% CI: 0.24–0.63) were inversely associated with statin use. These findings are detailed in Table 3, highlighting the demographic and clinical characteristics that influenced statin therapy prescription among the study population.

**Table 3:** Univariate analysis of factors associated with statin therapy for primary prevention of CVD before first STEMI.

Characteristic	Odds ratio	95% confidence interval	*p* value
Age, per 1‐year increase	1.06	1.02–1.10	0.002*
Sex (male)	0.20	0.07–0.56	0.002*
Race (Malay)	1.39	0.60–3.22	0.438
Diabetes	5.01	1.92–13.07	0.001*
Hypertension	19.26	5.53–67.06	< 0.001*
Dyslipidaemia	18.66	5.36–64.93	< 0.001*
Cerebrovascular disease	3.74	0.60–23.51	0.159
Chronic kidney disease	10.58	2.37–47.29	0.002*
History of smoking	0.29	0.12–0.67	0.004*
Family history of CVD	0.27	0.06–1.20	0.085
Baseline TC	0.53	0.35–0.81	0.003*
Baseline LDL‐C	0.39	0.24–0.63	< 0.001*

Abbreviations: CVD, cardiovascular disease; LDL‐C, low‐density lipoprotein cholesterol; TC, total cholesterol.

* *p* < 0.05 denotes statistical significance.

When these factors were analyzed using multivariate logistic regression analysis, only hypertension [adjusted odds ratio (AOR): 9.57; 95% CI: 1.86–49.28; *p* = 0.007)] and dyslipidaemia (AOR: 37.900; 95% CI: 6.72–213.87; *p* < 0.001) remained significantly associated with the use of statin therapy for the primary prevention of CVD prior to the first STEMI (Table 4).

**Table 4:** Multivariate analysis of factors associated with statin therapy for primary prevention of CVD before first STEMI.

Characteristics	AOR	95% CI	*p* value
Age, per 1‐year increase	1.06	0.99–1.14	0.103
Sex (male)	0.90	0.17–4.93	0.907
Diabetes	1.24	0.29–5.31	0.769
Hypertension	9.57	1.86–49.28	0.007*
Dyslipidaemia	37.90	6.72–213.87	< 0.001*
Chronic kidney disease	2.47	0.31–19.69	0.394
History of smoking	0.59	0.13–2.80	0.510
Baseline TC	0.89	0.22–3.57	0.865
Baseline LDL‐C	0.30	0.07–1.38	0.124

Abbreviations: AOR, adjusted odds ratio; CVD, cardiovascular disease; LDL‐C, low‐density lipoprotein cholesterol; TC, total cholesterol.

* *p* < 0.05 denotes statistical significance.

### 3.4 Cholesterol Levels ≥ 3 Months Post First STEMI

All patients were prescribed high‐intensity statin therapy (atorvastatin 40 mg once daily) upon discharge. Of these 93 patients had lipid profiles measured at least 3 months post‐STEMI. The mean TC level post‐STEMI was significantly lower compared to baseline levels (3.97 ± 1.10 mmol/L vs. 5.16 ± 1.17 mmol/L; *p* < 0.001). When stratified by baseline statin use, a significant reduction in mean TC levels post‐STEMI was observed only among patients without baseline statin therapy (3.91 ± 1.00 mmol/L vs. 5.28 ± 1.14 mmol/L; *p* < 0.001). In contrast, patients on baseline statin therapy showed no significant reduction in mean TC levels post‐STEMI (4.27 ± 1.51 mmol/L vs. 4.55 ± 1.20 mmol/L; *p* = 0.427).

Similarly, the mean LDL‐C level post‐STEMI was significantly lower than the baseline LDL‐C level (2.11 ± 0.89 mmol/L vs. 3.19 ± 1.07 mmol/L; *p* < 0.001). Stratified analysis revealed that patients without baseline statin use exhibited a significant reduction in mean LDL‐C level post‐STEMI (2.13 ± 0.81 mmol/L vs. 3.36 ± 0.99 mmol/L; *p* < 0.001). However, for patients on baseline statin therapy, the reduction in mean LDL‐C levels post‐STEMI was not significant (2.01 ± 1.25 mmol/L vs. 2.31 ± 1.11 mmol/L; *p* = 0.318).

When comparing lipid levels between patients with diabetes (*n* = 46) and patients without diabetes (*n* = 47) at least 3 months post‐STEMI, the mean TC level for patients with diabetes was 4.08 ± 1.22 mmol/L, while for those without diabetes, it was 3.86 ± 0.96 mmol/L. Similarly, the mean LDL‐C level for patients with diabetes was 2.08 ± 0.92 mmol/L compared to 2.14 ± 0.87 mmol/L for patients without diabetes. However, the differences in TC and LDL‐C levels between these two groups were not statistically significant (*p* > 0.05).

## 4 Discussion

According to the Malaysian CPG, patients with high or very high cardiovascular risk should aim for LDL‐C levels of ≤ 2.6 mmol/L and ≤ 1.8 mmol/L, respectively, or achieve a reduction of > 50% from baseline levels. This is typically achieved through a combination of statin therapy and lifestyle modifications [13]. Statin therapy has been shown to significantly reduce all‐cause mortality, major adverse cardiovascular events (MACE), and the need for revascularizations [14, 15], with an estimated 20%–30% reduction in CVD risk [14, 16, 17, 18].

This study highlights the low prescribing rate of statin therapy for primary prevention of CVD among patients prior to their first STEMI. Only 15.8% (*n* = 28) of patients received statin therapy before their first myocardial infarction, despite more than half of those without statins being classified as high CV risk based on Framingham General CVD Risk Score. This low utilization of statins for primary prevention may be partially attributed to lifestyle factors, as 65.8% of patients who did not receive statins were smokers. This suggests a potential correlation between unhealthy behaviors and lower adherence to preventive treatments, including statin therapy. Patients engaging in unhealthy behaviors may deprioritize their overall health management, underscoring the need for holistic lifestyle interventions alongside pharmacological therapy to effectively manage CV risk factors. Our findings align with previous studies reporting low rates of statin therapy among patients without prior CVD. For instance, a study by Mortensen and colleagues in Denmark found that only 16.0% of first MI patients without prior CVD were on statin therapy [19]. Another study found an even lower prevalence (10.8%) of statin therapy for primary prevention, though this study excluded patients with diabetes [17].

Among the patients who received statin therapy prior to their first STEMI, 89.3% (*n* = 25) were prescribed moderate‐intensity statins, despite more than half being classified as high CV risk. This prescribing pattern aligns with other studies investigating statin utilization in high CV risk populations, which report a predominant use of moderate‐intensity statins [9, 20, 21]. While moderate‐intensity statins can reduce LDL‐C levels, high‐intensity statins are more effective in lowering LDL‐C and reducing CV events, particularly in high‐risk individuals. Optimizing therapy by prescribing the maximum appropriate intensity of statins should be prioritized for patients with high CV risk, as recommended by clinical guidelines [22, 23, 24, 25]. These findings emphasize the significance of identifying high CV risk patients early using CVD risk assessment tool and prescribing tailored statin therapy to prevent future CVD events [26].

In terms of secondary prevention, our study found significantly lower TC and LDL‐C levels at least 3 months post‐STEMI compared to baseline levels, consistent with all patients being prescribed high‐intensity statin therapy (atorvastatin 40 mg daily) upon discharge. High‐intensity statins are estimated to reduce LDL‐C levels by at least 50% from baseline. Both local and international CPGs that recommend initiating high‐intensity statins (atorvastatin 40–80 mg daily or rosuvastatin 20–40 mg daily) as soon as possible following STEMI, regardless of baseline LDL‐C levels, and continuing therapy indefinitely [27, 28]. Additionally, achieving LDL‐C targets of < 1.8 mmol/L or at least a 50% reduction from baseline is strongly encouraged, with even lower LDL‐C goals (< 1.4 mmol/L) recommended for patients with very high CV risk, such as those with established [23, 27, 28, 29]. Notably, a similar pattern of statin prescribing was observed in a study conducted in Thailand, which reported a significant increase in the use of high‐intensity statins among patients with ACS, rising from 10% in 2013 to 88% in 2017 [30].

Although our study demonstrated high adherence to prescribing high‐intensity statin therapy post‐STEMI, the mean LDL‐C levels in our cohort were still above the guideline‐recommended targets [27, 28, 30, 31]. A prior local study evaluating lipid profiles post‐ACS reported even higher mean TC and LDL‐C levels post‐ACS (4.58 mmol/L and 2.75 mmol/L, respectively) which was likely attributable to the lower rate of high‐intensity statin prescriptions in that study (20.8%) [32]. However, this earlier study had a small sample size (*n* = 101) and was conducted in 2015, prior to the publication of updated guidelines recommending lower LDL‐C targets for secondary prevention [33].

Our analysis revealed that patients not on statins at baseline experienced significant reductions in mean LDL‐C and TC levels post‐STEMI, whereas those already on statins did not. This difference can be attributed to several factors. Patients already on baseline statinst end to have lower LDL‐C levels due to the cholesterol‐lowering effects of the medication, leading to a plateau effect where additional reductions are less pronounced post‐STEMI. Conversely, patients without prior statin use typically have higher baseline LDL‐C levels, allowing for greater reductions when high‐intensity statins are initiated. Statins enhance LDL clearance by upregulating LDL receptors on hepatocytes, a mechanism particularly impactful when initiated post‐STEMI in statin‐naïve patients. These patients experience a substantial increase in receptor activity, leading to significant LDL‐C reductions. In contrast, chronic statin users have already maximized receptor upregulation, limiting further LDL‐C lowering. Additionally, the acute phase response following STEMI temporarily disrupts lipid metabolism, with statin‐naïve patients often showing a more pronounced response to intensive statin therapy as their lipid levels stabilize post‐treatment.

Despite these findings, achieving guideline‐recommended LDL‐C targets often requires statin dose titration to the maximally tolerated level. If LDL‐C levels remain suboptimal, combination therapy with additional lipid‐lowering agents, such as ezetimibe or PCSK9 inhibitors, may be necessary. Nevertheless, many patients with suboptimal LDL‐C levels fail to receive statin intensification or modifications to their lipid‐lowering regimen modifications, even under cardiologist care [34]. High‐intensity statin therapy alone is often insufficient to fully mitigate CVD risk [35]. Regular lipid monitoring and timely up‐titration to higher statin doses are critical, as these interventions are strongly associated with a reduced risk of MACE, particularly in high‐risk patients who benefit the most from aggressive lipid‐lowering strategies [36].

This study has several limitations that should be acknowledged. First, the small sample size of patients on statins prior to STEMI limits the generalizability of our findings. Second, the retrospective design of the study may introduce bias due to the potential for incomplete or inaccurate documentation in the medical records. Third, lifestyle factors, particularly the high prevalance of smoking among the study population, may act as confounders, influencing the relationship between statin use and cardiovascular outcomes. Finally, as the study was conducted in a single secondary hospital in Malaysia, the applicability of the findings to other populations or healthcare settings may be limited.

## 5 Conclusion

In conclusion, our study highlights a low rate of statin prescribing for the primary prevention of CVD among patients in Malaysia prior to their first STEMI. Hypertension and dyslipidemia were identified as the only significant factors associated with statin use. Furthermore, while TC and LDL‐C levels were significantly reduced during follow‐up post‐STEMI, the achieved LDL‐C levels often failed to meet recommended targets, reflecting a gap in statin dose intensification and lipid‐lowering strategies. These findings underscore the critical need for enhanced efforts in both primary and secondary CVD prevention, including early identification of high‐risk patients and the implementation of more aggressive lipid‐lowering interventions to better align with clinical guidelines.

## Author Contributions

**Abdullah Faiz Zaihan:** conceptualization, investigation, writing–original draft, methodology, writing–review & editing, formal analysis. **Shairyzah Ahmad Hisham:** supervision, conceptualization, methodology, formal analysis, data curation. **Aina Yazrin Ali Nasiruddin:** conceptualization, methodology, formal analysis, data curation, supervision. **Chia Siang Kow:** conceptualization, formal analysis, writing–review & editing; data curation. **Syed Shahzad Hasan:** writing–review & editing, formal analysis, data curation. **Dinesh Sangarran Ramachandram:** writing–review & editing, formal analysis, data curation, conceptualization.

## Ethics Statement

The study was retrospectively conducted and the data collected were aggregated, therefore, patient consent was not necessary. The study was registered with the National Medical Research Register (NMRR) under the reference number NMRR ID‐22‐01623‐ZHX and was ethically approved by the Medical Research and Ethics Committee (MREC).

## Conflicts of Interest

The authors declare no conflicts of interest.

## Transparency Statement

The lead author Abdullah Faiz Zaihan affirms that this manuscript is an honest, accurate, and transparent account of the study being reported; that no important aspects of the study have been omitted; and that any discrepancies from the study as planned (and, if relevant, registered) have been explained.

## Data Availability

The data that support the findings of this study are available from the corresponding author upon reasonable request.
